# A novel zinc finger transcription factor, BcMsn2, is involved in growth, development, and virulence in *Botrytis cinerea*

**DOI:** 10.3389/fmicb.2023.1247072

**Published:** 2023-10-17

**Authors:** Ping Lu, Ke Wang, Jiaqi Wang, Chunbo Xia, Shu Yang, Liang Ma, Haojie Shi

**Affiliations:** ^1^The Key Laboratory for Quality Improvement of Agricultural Products of Zhejiang Province, College of Advanced Agricultural Sciences, Zhejiang A&F University, Hangzhou, China; ^2^National-Regional Joint Engineering Research Center for Soil Pollution Control and Remediation in South China, Guangdong Key Laboratory of Integrated Agro-environmental Pollution Control and Management, Institute of Eco-environmental and Soil Sciences, Guangdong Academy of Sciences, Guangzhou, China

**Keywords:** *Botrytis cinerea*, zinc finger protein, ROS, transcriptional regulator, RNA-Seq

## Abstract

Reactive oxygen species (ROS) are important for plant defense against fungal attack. As a necrotrophic fungus, *Botrytis cinerea* can exploit ROS that originated from both sides of the host and pathogen during interaction to facilitate its infestation. Meanwhile, *B. cinerea* needs to exert an efficient oxidative stress responsive system to balance the intracellular redox state when encountering deleterious ROS levels. However, the machinery applied by *B. cinerea* to cope with ROS remains obscure. Herein, we investigated the role of the transcription factor BcMsn2 in regulating *B. cinerea* redox homeostasis. Disruption of the *BcMsn2* gene severely impaired vegetative growth, sclerotium formation, conidial yield, and fungal virulence. The intracellular oxidative homeostasis of the ∆*bcmsn2* mutant was disrupted, leading to significantly elevated levels of ROS and reduced activities of enzymes closely associated with oxygen stress, such as catalase (CAT) and superoxide dismutase (SOD). RNA-Seq and qRT-PCR analyses showed remarkable downregulation of the expression of several genes encoding ROS scavenging factors involved in maintaining the redox homeostasis in ∆*bcmsn2*, suggesting that BcMsn2 functions as a transcriptional regulator of these genes. Our findings indicated that BcMsn2 plays an indispensable role in maintaining the equilibrium of the redox state in *B. cinerea*, and intracellular ROS serve as signaling molecules that regulate the growth, asexual reproduction, and virulence of this pathogen.

## 1. Introduction

*Botrytis cinerea* is a necrotrophic ascomycete fungus that can infect over 1,400 species, causing massive economic losses worldwide annually (Williamson et al., 2007). *Botrytis cinerea* has a broad host range, is aggressive, and causes substantial damage; therefore, it is considered as one of the most important plant pathogens by fungal plant pathologists (Dean et al., 2012). The life cycle of *B. cinerea* has been extensively studied, revealing a predominantly asexual mode of reproduction. The production of conidia is regulated by the external environment and precisely regulated by the circadian system in cells (Martinez et al., 2003; Yoshida et al., 2011; Cohrs et al., 2016; Rascle et al., 2018). Previous studies have identified several genes encoding proteins involved in conidiation, including light-responsive transcriptional regulator genes *BcREG1* and *BcLTF3* (Michielse et al., 2011; Brandhoff et al., 2017), genes encoding components of the cAMP-dependent pathway (*BCG1*, *BCG3*, and *BcPKA1*) (Doehlemann et al., 2006; Williamson et al., 2007; Schumacher et al., 2008), the gene encoding the Gβ-subunit of the heterotrimeric G protein, *BcGB1* (Williamson et al., 2007; Tang et al., 2021), and the MAP kinase-encoding genes *BcBMP1* and *BcSAK1* (Doehlemann et al., 2006; Segmüller et al., 2007). Therefore, understanding the growth, conidiation, and pathogenic mechanisms of *B. cinerea* is key to the prevention and control of gray mold.

Reactive oxygen species (ROS) are small molecules, including hydrogen peroxide (H_2_O_2_), the superoxide anion radical (O_2_^−^), and the hydroxyl radical (OH), which exhibit high oxidative activity. They are generated by plants and pathogens during the infection stage, and are also produced continuously as byproducts of various metabolic pathways in cellular components, such as mitochondria, peroxisomes, and chloroplasts, with mitochondria being the main source of intracellular ROS (Viefhues et al., 2014). Excess intracellular ROS can damage macromolecules and even lead to cell death. Phytopathogenic fungi have developed sophisticated defense systems to maintain redox homeostasis. Enzymatic ROS scavenging mechanisms involve various peroxidases, such as peroxidase (POD), superoxide dismutase (SOD), and catalase (CAT). However, ROS can also act as signaling molecules to regulate growth, sporulation, and virulence. The generation and scavenging of ROS exhibit circadian rhythms, a fundamental biological phenomenon observed in various species that plays a direct role in many physiological processes (Mailloux, 2015; Fanjul-Moles and López-Riquelme, 2016; Milkovic et al., 2019). A certain range of ROS can promote sclerotial differentiation and conidia germination in *Sclerotinia sclerotiorum* (Georgiou et al., 2000; Papapostolou et al., 2014). In *Neurospora crassa*, the ROS oscillation observed under free-running conditions might be produced by *Cat1* [6]. In *B. cinerea*, the circadian oscillator BcFRQ1 plays a crucial role in conidiation, sclerotia formation, and virulence (Hevia et al., 2015).

In *Saccharomyces cerevisiae*, regulation of ROS is associated with the rapid induction of at least 115 proteins, such as Yap1, Skn7, and Msn2/4 (Kuge and Jones, 1994; Morgan, 1997; Godon et al., 1998; Charizanis et al., 1999; Lee et al., 1999). Msn2/4 directly induces the expression of genes encoding antioxidant enzymes, such as catalase, superoxide dismutases, and peroxidases (Hasan et al., 2002; Drakulic et al., 2005). Orthologs of *Msn2* have been characterized in several fungi, including *Aspergillus parasiticus*, *Magnaporthe oryzae*, *Candida albicans*, and *Sclerotinia sclerotiorum*. The *msnA* deletion strains of *A. parasiticus* and *A. flavus* exhibited retarded colony growth with increased conidiation and elevated levels of ROS (Chang et al., 2011). *MoMsn2* in *M. oryzae* is required for aerial hyphal growth and conidiation (Zhang et al., 2014).

In this study, a mutation in BcMsn2, a novel zinc finger transcription factor, resulted in severely retarded vegetative growth, significantly delayed sclerotium formation, a remarkable decrease in conidial yield, and defects in host plant infection. However, the mechanisms underlying the functions of BcMsn2 remain unknown. Herein, we found that *BcMsn2* deletion mutants exhibited increased intracellular levels of ROS, leading to irreversible disruption of the cellular redox state. The activities of enzymes closely related to oxygen stress, such as CAT and SOD, were reduced. Additionally, using RNA sequencing (RNA-seq) and quantitative real-time reverse transcription PCR (qRT-PCR) analyses, we observed significant downregulation of several ROS scavenging genes involved in maintaining the redox homeostasis in the *∆bcmsn2* mutant. These findings indicated that BcMsn2 might function by maintaining the redox equilibrium, and intracellular ROS might act as second messengers to regulate growth, asexual reproduction, and virulence in *B. cinerea*.

## 2. Materials and methods

### 2.1. Strains, media, and growth conditions

The wild-type *B. cinerea* strain B05.10 was used for the transformation experiments in this study (van Kan et al., 1997). Strain B05.10 was cultured on potato dextrose agar (PDA) plates. Potato dextrose broth (PDB) served as the liquid medium for mycelium collection. The *BcMsn2* gene deletion mutants and complemented strains were maintained on PDA supplemented with 100 μg/mL hygromycin B (Sigma, St. Louis, MO, United States) or 100 μg/mL nourseothricin (Jena Bioscience, Jena, Germany), respectively. SH agar medium containing 0.6 M sucrose, 4 mM Tris–HCl, 1 mM (NH_4_)_2_HPO_4_, and 1.2% agar was used to regenerate *B. cinerea* protoplasts.

### 2.2. Generation of the BcMsn2 deletion and complementation mutants

The sequence of *BcMsn2* (Gene ID: Bcin01g05160) was obtained from the Ensembl fungi.^1^ To investigate the biological function of BcMsn2 in *B. cinerea*, we generated *BcMsn2* deletion mutants through protoplast formation and transformation (Gronover et al., 2001).

The flanking sequences of the *BcMsn2* gene were amplified from B05.10 genomic DNA using the primer pair P1/P2 and P5/P6 (all primers are shown in Table S1). The sequence of the gene encoding hygromycin B phosphotransferase (HPH) was amplified from the pBS-HPH1 vector using the primer pair P3/P4. The gene replacement construct was created by double-joint PCR (DJ-PCR) (Yu et al., 2004) (Supplementary Figure S1A). Three deletion mutants, *∆bcmsn2-1a1*, *∆bcmsn2-3b1*, and *∆bcmsn2-4a1* were identified among 50 hygromycin-resistant transformants using PCR analysis with the primers Gene-F and Gene-R (Supplementary Figure S1B and Supplementary Table S1). All deletion mutants showed identical phenotypic characteristics.

The *BcMsn2* deletion mutants *∆bcmsn2-3b1* and *∆bcmsn2-4a1* were complemented with the complete *BcMsn2* gene. To construct the p1300-NAT1 vector, the *NAT1* gene was amplified using primers P13/P14 from the pD-NAT1 vector (Kuck and Hoff, 2006). The PCR product was cloned into the *Xho*I site of p1300BAR. The complemented *BcMsn2* gene was amplified from the genome of the parental strain B05.10 using the P9/P10 primer pair. The resulting PCR product was cloned into the *Eco*RI-*Bam*HI sites of p1300-NAT1 to generate the complementation vector, p1300-BcMsn2-C. *BcMsn2* in this vector was sequenced to verify that no errors were present in this sequence before transformation into the *BcMsn2* deletion mutant using *Agrobacterium tumefaciens*-mediated transformation (*At*MT) (Rolland et al., 2003). The hygromycin-resistant and nourseothricin-resistant *BcMsn2* complemented mutants were identified using PCR analysis with the primers Gene-F and Gene-R. The transformant *∆bcmsn2*-C that had approximately similar expression levels of the *BcMsn2* gene to the wild-type B05.10 strain was selected by qPCR.

### 2.3. Yeast complementation assay

The *BcMsn2* gene was amplified with primers pYES2-F/pYES2-R and cloned into the *Hind*III-*Bam*HI sites of the yeast expression vector pYES2, generating the vector pYES2-BcMsn2. Transformation of pYES2-BcMsn2 into a *∆msn2* mutant of *S. cerevisiae* was carried out using the lithium acetate method (Schiestl and Gietz, 1989). The yeast transformants were grown on YPRG medium (1% yeast extract, 2% peptone, 1% galactose, 1% raffinose, 1.5% agar). The wild-type strain BY4741, the *∆scmsn2* mutant, and the *∆scmsn2* mutant transformed with empty pYES2 vector were used as controls.

### 2.4. Study of the conidiation, hyphal growth and sclerotia, and assays of pathogenicity

Hyphae of the wild-type strain, *BcMsn2* deletion mutants, and complemented strains were incubated on PDA plates at 22°C for 2 days. A mycelial agar plug (5 mm in diameter) was cut and transferred onto the center of a fresh PDA plate. The plates were incubated at 22°C. Radial growth was determined by measuring the colony diameter daily for 5 consecutive days. Sclerotia production was observed for 1 month under dark conditions at 22°C.

For the conidiation assay, conidia numbers were counted after 3, 5, and 7 days of incubation on PDA plates. Seven-day-old conidia were also used for size statistics and germination experiments in GB5 medium (Guan et al., 2020), At least 100 conidia were counted per replicate in each experiment. Three replicates were performed for each conidia age.

For the pathogenicity assays, the leaves of beans, and the fruits of tomato, pear, and apple were inoculated with mycelial plugs (2-day-old, 5 mm in diameter) of each strain. Inoculated materials were kept in Petri dishes with high humidity at 22°C for 3 days, and the lesion diameters were measured daily.

### 2.5. Assays of ROS and antioxidant enzyme activity

For ROS detection using nitroblue tetrazolium (NBT) staining, plates with three-day-old cultures of each strain were stained with 0.05% NBT, and the reaction was terminated using anhydrous ethanol after 1 h (Jing et al., 2020). The staining results were observed under a stereomicroscope. ROS detection using 2′, 7’-Dichlorodihydrofluorescein diacetate (H_2_DCF-DA) was performed using Reactive Oxygen Species Assay Kit (Code No. S0033S, Beyotime Biotechnology, Jiangsu, China).

The conidia suspension at a final concentration of 1 × 10^4^ was added to 50 mL of PDB medium and incubated in a shaker for 3 days. The mycelia of each strain were collected, weighed, and 2.0 g of mycelium was added to 10 mL PBS [0.1 M, pH 7.4, containing 3% polyvinylpyrrolidone (PVP)]. It was then ground into a 10% tissue homogenate, and the crude extract was obtained by centrifugation at 4000 rpm, 4°C. H_2_O_2_, CAT, POD, SOD, and malondialdehyde (MDA), used to indicate enzyme activity, were measured using kits (Nanjing Jiancheng Bioengineering Institute, Nanjing, China).

### 2.6. Detection membrane potential of mitochondria

Mitochondrial membrane potential was detected with Enhanced mitochondrial membrane potential assay kit with JC-1 (Beyotime Biotechnology, C2003S). The final concentration of 1 × 10^6^ conidia suspensions of *B. cinerea* were placed in 50 mL PDB medium and incubated in a shaker for 12 h. 1 mL suspensions were centrifuged at 8000 rpm for 5 min, then the precipitates were resuspended in 500 μL JC-1 (1×) and incubated in dark place for 20 min at 37°C. Centrifuge and resuspend the precipitate in 500 μL PBS to remove the excessive JC-1. Green fluorescence and red fluorescence were observed using the laser scanning confocal microscope. Fluorescence intensities were counted using ImageJ software.

### 2.7. RNA-Seq analysis

Mycelia of the wild-type strain B05.10 and *∆bcmsn2* were collected from 10-h PDA cultures. Total RNA samples were extracted using the High-Salt Solution for Precipitation (Plant) kit (Code No.9193, Takara, Dalian, China). Two biological replicates were prepared for each strain. Library construction and sequencing were performed to generate 50 bp reads. The RNA-Seq reads were mapped to the reference genome (ASM83294v1, Ensembl fungi v53; ENA accession number GCA_000143535) using HISAT2 (Pertea et al., 2016). The mapping statistics are shown in Supplementary Table S2. Differentially expressed transcripts were identified using DESeq2 (Love et al., 2014) based on transcript abundance calculated by Salmon (Patro et al., 2017). Transcripts with |log2 fold change| ≥ 1 and adjusted value of *p* ≤0.05 were considered differentially expressed. The data generated in this project have been deposited in the NCBI database with accession numbers SRR23730092–SRR23730095.

### 2.8. qRT-PCR analysis

The same amounts of conidia of different strains were inoculated into PDB medium and cultured in a shaker at 22°C and 180 rpm for 2 days. Total RNA samples of the wild-type B05.10 and deletion mutants were utilized for genomic DNA (gDNA)-free cDNA synthesis using the PrimeScript™ RT reagent Kit with gDNA Eraser (Code No. RR047A, Takara). Quantitative real-time PCR amplification of the cDNA was carried out using the BIO-RAD Real-Time PCR Detection System (Bio-Rad, Hercules, CA, United States), with the TB Green® Premix Ex Taq™ (Code No. RR420A, Takara). The *GAPDH* gene (encoding glyceraldehyde-3-phosphate dehydrogenase) was used as a reference gene to normalize the target gene expression and correct for sample-to-sample variation (Ren et al., 2017). The relative transcript abundances of selected transcripts were calculated using the 2^−∆∆Ct^ method from the mean of three independent determinations of the threshold cycle (Livak and Schmittgen, 2001). The primers used in this study are shown in Supplementary Table S1.

## 3. Results

### 3.1. Identification and deletion of BcMsn2 in *Botrytis cinerea*

**Figure 1:**
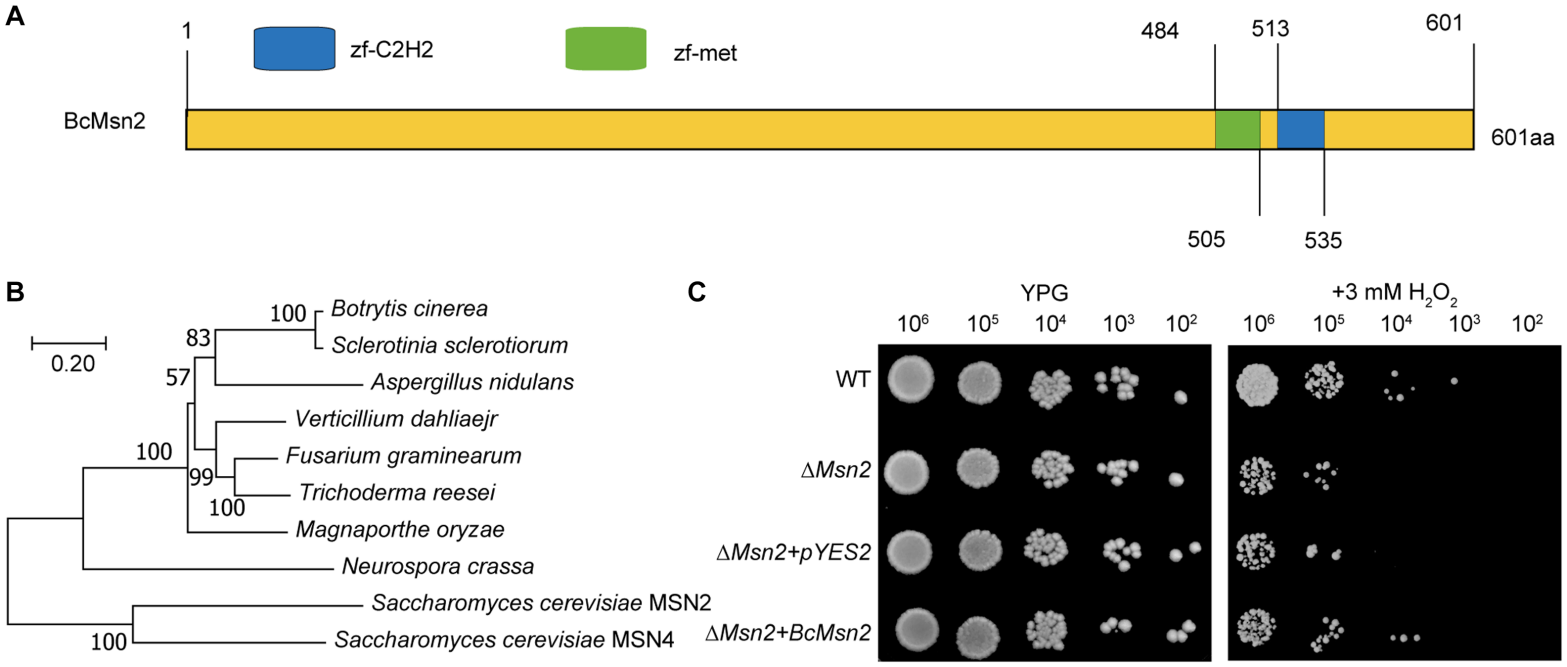
Sequence and phylogenetic analysis of Msn2. **(A)** Sequence analysis of BcMsn2 in *B. cinerea*. Zf-C2H2: A zinc finger is part of a protein that can bind to DNA. zf-met: A zinc-finger domain with the CxxCx(12)Hx(6)H motif. **(B)** The phylogenetic tree of Msn2 with homologs gene from other fungal species was constructed using MEGA version 11. Numbers at the nodes in the rooted tree represent the bootstrap value after 1000 replications. The bar indicates 0.20 distance units. **(C)** Yeast cells (10^2^ to 10^6^ cells per milliliter) of WT, ∆*scmsn2* mutant, and ∆*scmsn2*/*BcMsn2* or ∆*scmsn2*/pYES2 transformants were assayed for growth on 1% yeast extract, 2% peptone, 3% glycerol (galactose) plates, with or without 3 mM H_2_O_2_.

In this study, we identified a novel protein named as BcMsn2 (Gene ID: Bcin01p05160) in *B. cinerea*. Sequence analysis confirmed that the coding region of *BcMsn2* gene has one intron and two exons, and the coding sequence (CDS) comprises 1806 bp, encoding a polypeptide of 601 amino acid, consistent with the annotation in the genome database. The predicted BcMsn2 protein shared 66.6% identity with its *S. cerevisiae* counterpart MSN2. Analysis of BcMsn2 in the Conserved Domain Database revealed the presence of two conserved C_2_H_2_ zinc finger DNA-binding domains located at amino acid positions 484–535, characterized by the following pattern [#-X-C-X(1–5)-C-X3-#-X5-#-X2-H-X(3–6)-(H/C)] (Figure 1A). Phylogenetic analysis showed that BcMsn2 was most closely related to the MSN2 protein from *S. sclerotiorum* (Figure 1B). To investigate the function of *BcMsn2*, gene deletion mutants were generated via homologous recombination (Supplementary Figure S1A), and the hygromycin-resistant transformants were initially screened using PCR analysis (Supplementary Figure S1B). The *BcMsn2* deletion mutant was complemented by introducing the complete *BcMsn2* gene using *At*MT, and the nourseothricin-resistant transformants were confirmed by PCR (Figure S1B). Additionally, to assess the functionality of *BcMsn2* in *S. cerevisiae*, the open reading frame of *BcMsn2* was cloned into the pYES2 vector and transformed into a ∆*msn2* mutant of *S. cerevisiae*, which is known to be hypersensitive to oxidative stress. This transformation excluded any potential interference from the empty pYES2 vector (Figure 1C). The yeast transformants containing the pYES2-BcMsn2 construct exhibited restored growth of the ∆*scmsn2* mutant on medium supplemented with 3 mM H_2_O_2_ (Figure 1C). These findings indicated that BcMsn2 possesses a conserved function in the oxidative stress response pathway.

### 3.2. BcMsn2 plays a critical role in cellular ROS balance

**Figure 2:**
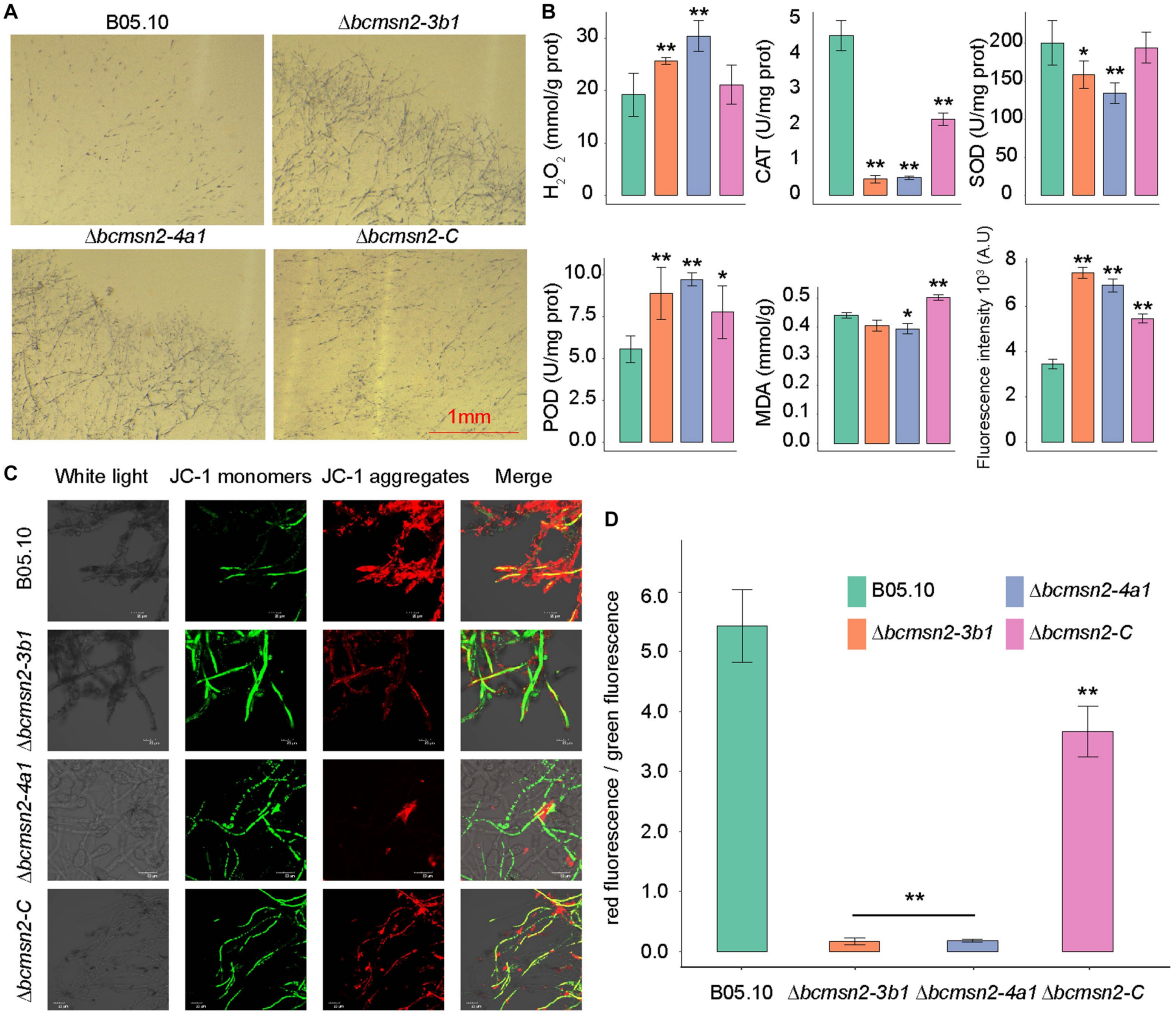
∆*bcmsn2* displayed a higher H_2_O_2_ content, increased POD activity, decreased CAT and POD activities, higher ROS levels, and lower mitochondrial membrane potential than the WT and ∆*bcmsn2-C*. **(A)** Each strain at 3 days old was stained with 0.05% NBT. Bar = 1 mm. **(B)** Comparisons of H_2_O_2_, CAT, SOD, POD, MDA, and ROS among the WT, ∆*bcmsn2*, and ∆*bcmsn2-C* after incubation in PDB for 4 days. (*p< 0.05, **p < 0.01). **(C)** Detection of mitochondrial membrane potential using the laser scanning confocal microscope. **(D)** Relative ratio of red/green fluorescence after JC-1 dye.

To investigate the involvement of *BcMsn2* in maintaining the ROS balance, we measured the H_2_O_2_ content in the ∆*bcmsn2* mutants and observed a 35–50% increase compared with that in the wild type (WT) and ∆*bcmsn2-C* strains. We assessed O_2_^·-^ levels by using NBT staining, and similar to H_2_O_2_, we observed a significant accumulation of O_2_^·-^ in the ∆*bcmsn2* mycelium (Figure 2A). Furthermore, to detect ROS, we stained the conidia using H_2_DCF-DA. The conidial ROS content in the ∆*bcmsn2* mutant was 200% higher than that of the WT, which is consistent with the previously measured elevated H_2_O_2_ and O_2_^-^ levels (Figure 2B). The enzyme activities of CAT, POD, and SOD were determined in each strain. The results demonstrated a significant decrease in CAT and SOD activities, while POD activity was increased in the ∆*bcmsn2* mutant (Figure 2B). Further research found the mitochondrial membrane potential of the two mutants significantly decreased and were subjected to more mitochondrial damage. Changes in membrane is a central feature of mitochondrial health. Mitochondrial membrane potential depolarization is a good indicator to evaluate mitochondrial dysfunction. When the mitochondrial membrane potential is high, JC-1 forms J-aggregates that produce red fluorescence; when the potential is low, JC-1 is a monomer that produces green fluorescence. Changes in mitochondrial membrane potential were detected by red and green fluorescence transformation. The proportion of mitochondrial depolarization is measured by the relative proportion of red and green fluorescence. We detected membrane potential of mitochondria by JC-1 staining and found more green fluorescence and a lower relative ratio of red/green fluorescence in the two mutants (∆*bcmsn2-3b1* and ∆*bcmsn2-4a1*) (Figures 2C,D). These findings indicated the ∆*bcmsn2* mutants have higher intracellular ROS level that damage mitochondrial membranes.

### 3.3. Effect of BcMsn2 deletion on vegetative growth and reproductive differentiation

**Figure 3:**
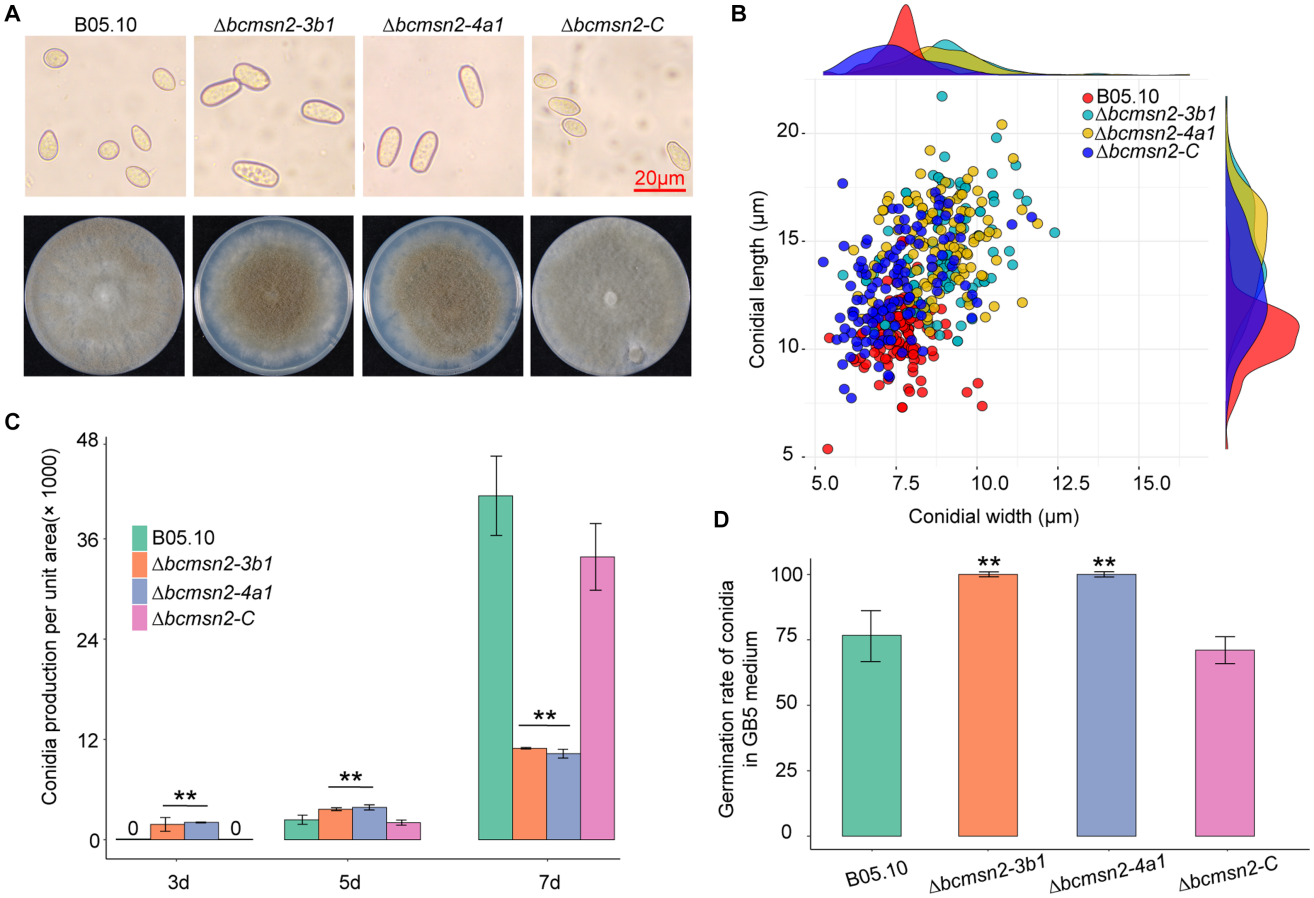
The effect of *BcMsn2* knockout on spore morphology, germination, and conidia production. **(A)** Conidia of the *∆bcmsn2* mutants displayed abnormal morphology with elongation observed under the microscope Bar = 20 mm. The conidial layer was also more concentrated in the 7-day-old colonies. **(B)** Conidia length and width statistics. **(C)** The conidia production of the strains cultured on PDA plates was investigated at 3, 5, and 7 days. **(D)** The conidial germination rate of the strains cultured on GB5 was investigated at 4 hours. (*p < 0.05, **p < 0.01).

To test whether BcMsn2 is involved in conidial morphogenesis, we determined the ability of each strain to produce conidia at 3, 5, and 7 dpi on PDA plates. Analysis of conidial morphology revealed that the conidia produced by ∆*bcmsn2* were significantly different from those of WT in length and width, with a 39% increase in length (10.60 ± 1.46 μm, 14.73 ± 2.08 μm, and 12.83 ± 1.17 μm for conidial length produced by the WT, ∆*bcmsn2*, and ∆*bcmsn2-C* strains, respectively) and an 18% increase in width (7.66 ± 0.75 μm, 9.03 ± 1.22 μm, and 7.48 ± 1.17 μm for conidial width produced by the WT, ∆*bcmsn2*, and ∆*bcmsn2-C* strains, respectively) (Figures 3A,B). These results suggested that the loss of *BcMsn2* in *B. cinerea* increased the conidial size of the pathogen. In addition, our data showed that the conidia produced by ∆*bcmsn2* could be collected at 3 dpi, whereas the WT conidia could be collected at 5 dpi. However, the total number of conidia per unit area of ∆*bcmsn2* at 7 dpi was only 24% of that of the wild type strain (4.43 × 10^5^ ± 3.36 × 10^4^ cm^−1^, 1.06 × 10^5^ ± 3.94 × 10^4^ cm^−1^, 3.94 × 10^5^ ± 3.28 × 10^4^ cm^−1^ for conidia production per unit area by the WT, ∆*bcmsn2*, and ∆*bcmsn2-C* strains, respectively) (Figure 3C). When incubated on GB5 medium, approximately 75% of the B05.10 conidia germinated at 4 h. Under the same conditions, the germination rate of ∆*bcmsn2* conidia was 98% (Figure 3D). Overall, our results demonstrate that BcMsn2 controls conidial morphogenesis and is dispensable for the radial and lateral growth of conidiation in *B. cinerea*.

**Figure 4:**
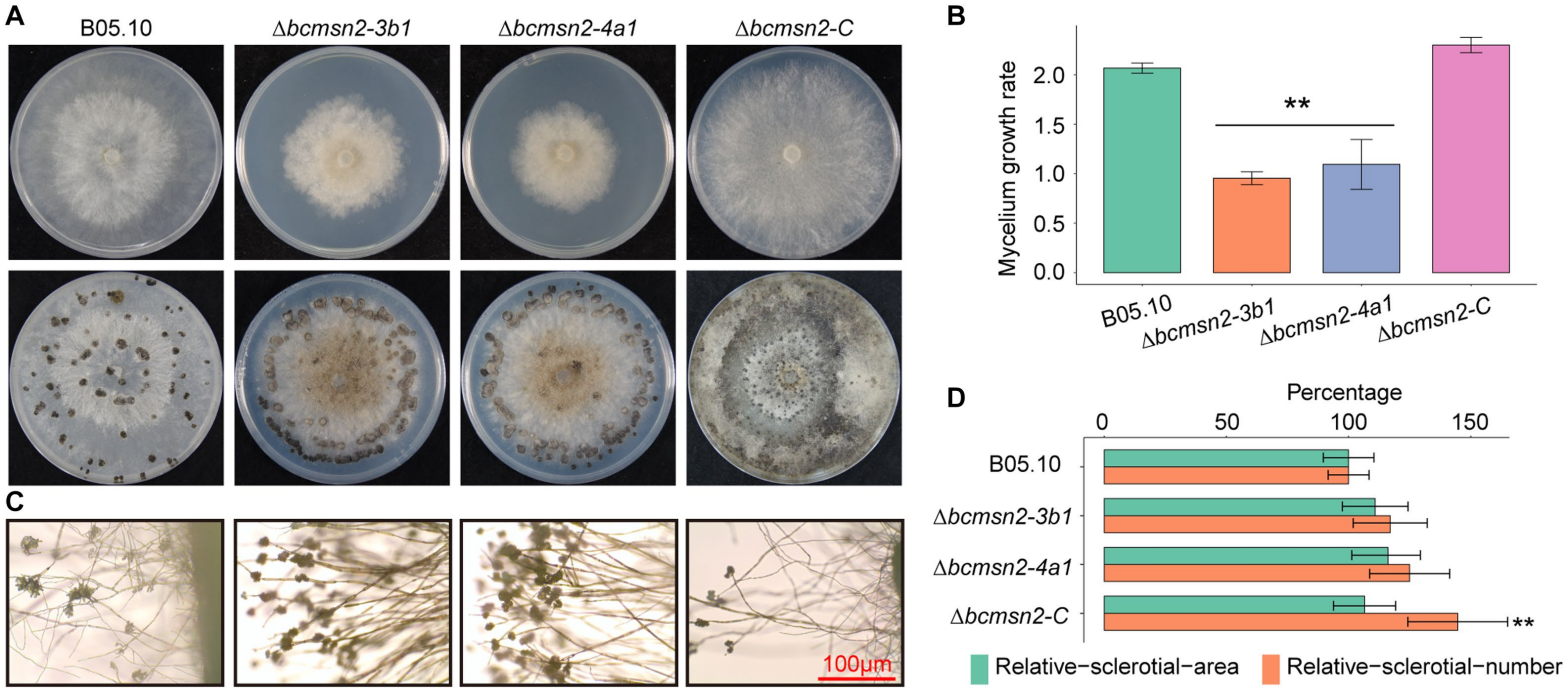
BcMsn2 is required for hyphal growth and conidiation in *B. cinerea*. **(A)** BcMsn2 mediates *B. cinerea* aerial mycelium (upper panel) and sclerotia (lower panel) production. Aerial mycelium and sclerotia of the wild-type (WT), ∆*bcmsn2*, and complemented strains were inoculated on PDA plates at 20°C for 4 days and 35 days in the dark, respectively. **(B)** The mycelium growth rate on PDA plates at 20°C for 3 days. Error bars represent the standard deviation and asterisks represent the significant difference (*p < 0.05, **p < 0.01). **(C)** Conidial and conidiophore formation were observed under a light microscope. Bar = 100 μm. **(D)** Quantification of the number and area of sclerotia.

To determine the function of BcMsn2 in hyphal growth, ∆*bcmsn2* and the complementary strain ∆*bcmsn2-C* were cultured on the PDA media for 4 days, after which the colony morphology was observed. The results demonstrated that the ∆*bcmsn2* mutants showed a decreased colony diameter compared with that of the WT (Figure 4A). The colony diameter (at 4 days post inoculation (dpi)) and the mycelium growth rate (at 3 dpi) were reduced by approximately 53% (Figure 4B). Compared with the WT, ∆*bcmsn2* showed increased conidiophore formation (Figure 4C). To investigate the role of BcMsn2 in sclerotia formation, ∆*bcmsn2* and the complementary strain ∆*bcmsn2-C* were cultured on the PDA media for 35 days in the dark. The results demonstrated that the formation of sclerotia was dramatically delayed by 14 days in ∆*bcmsn2* compared with that in B05.10. The relative area and number of sclerotia increased after culturing on PDA media for one month; however, the difference among the strains was not significant (Figures 4A,D). Overall, these results demonstrated that loss of *BcMsn2* in *B. cinerea* effected hyphal growth and sclerotia formation.

### 3.4. BcMsn2 plays a critical role in virulence

**Figure 5:**
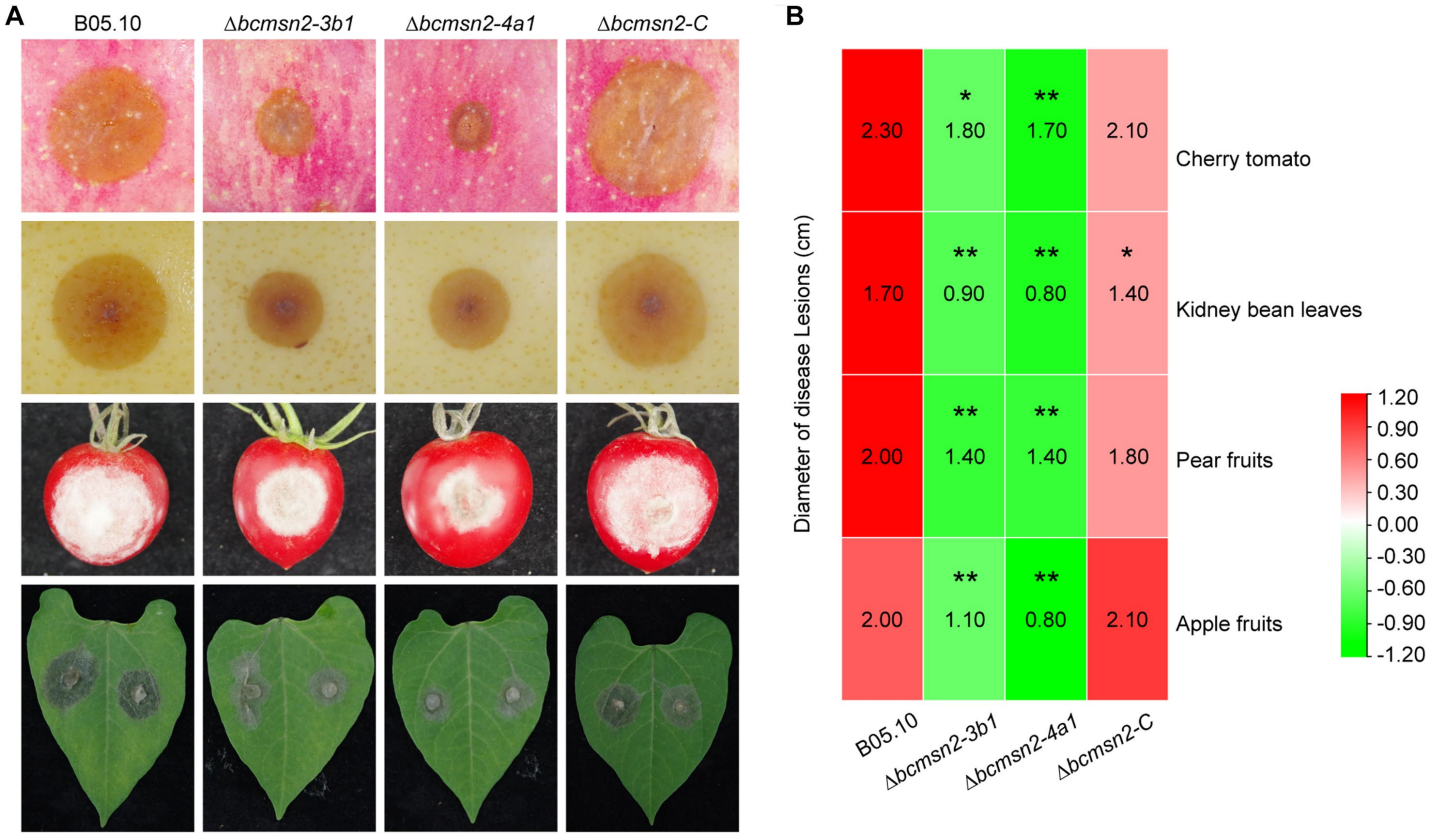
∆*bcmsn2* displayed lower virulence. **(A)** Pathogenicity assays of each strain on the leaves of bean, and the fruits of tomato, pear, and apple. Disease symptoms were photographed at 60 h post-inoculation (hpi). **(B)** The diameters of disease lesions of each strain shown in **(A)**. (*p < 0.05, **p < 0.01).

To determine the role of BcMsn2 in *B. cinerea* pathogenicity, infection assays were conducted using different plant tissues (the leaves of beans, the fruits of tomato, pear, and apple), which serve as entry points for mycelia (Figure 5A). At 60 h post-inoculation (hpi), the lesion diameters of ∆*bcmsn2* mutants on apple pear, and tomato fruits, and bean leaves were reduced by 53, 30, 24, and 50%, respectively, compared with those of WT (Figure 5B). These data suggested that BcMsn2 plays an important role in the invasive growth of *B. cinerea* in planta.

### 3.5. BcMsn2 regulates cellular ROS scavenging genes

**Figure 6:**
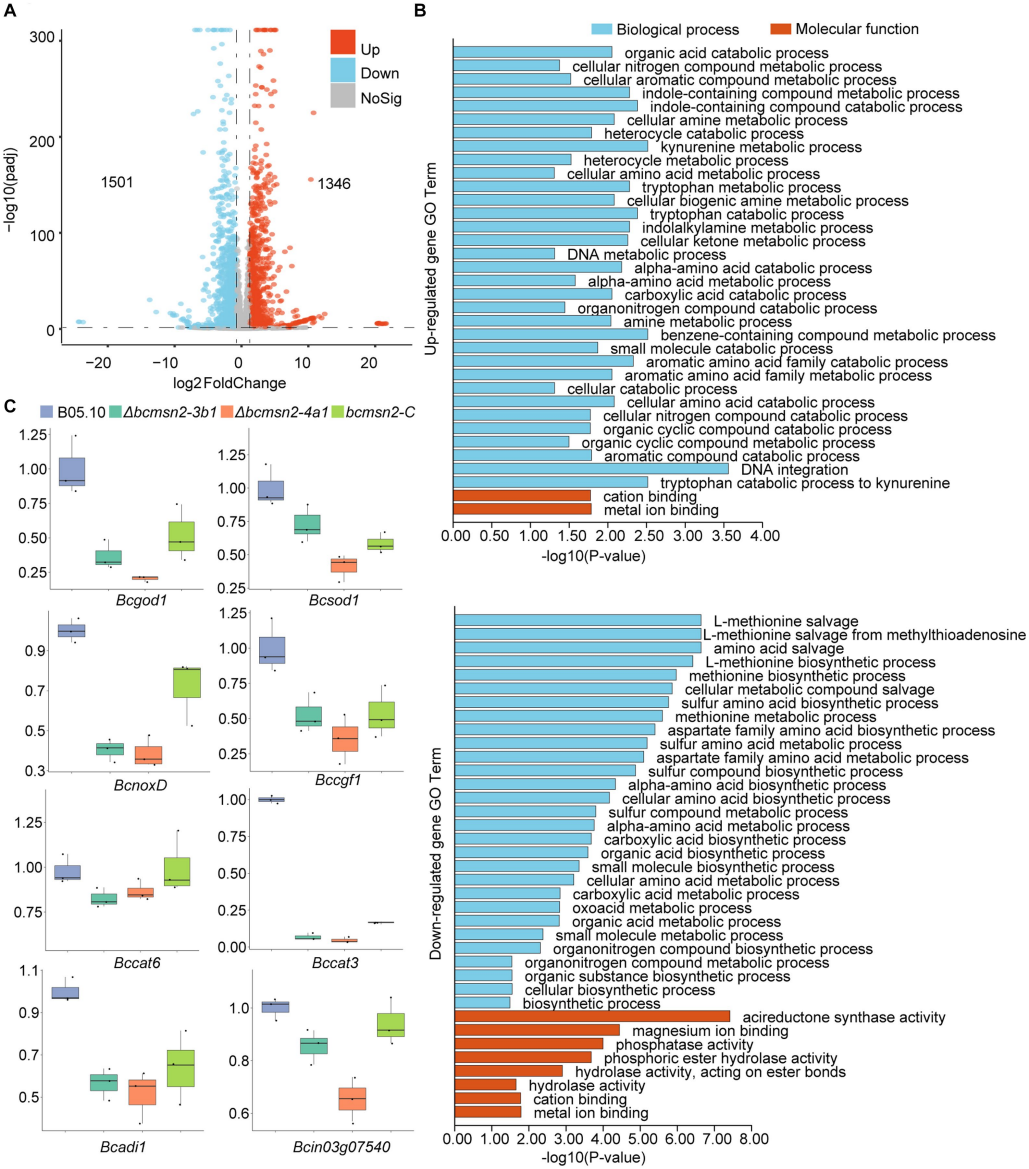
RNA-Seq analysis of the ∆*bcmsn2* strain. **(A)** Volcano plot of the significantly upregulated and downregulated genes in the ∆*bcmsn2* mutant (|log2 Fold Change| ≥ 1, *p*-value <0.05) compared with those in the wild-type. The numbers of differentially expressed genes were calculated with data from two biological replicates. **(B)** Enriched gene ontology (GO) terms associated with the upregulated and downregulated genes in the ∆*bcmsn2* mutants. **(C)** qRT-PCR detection of the expression of ROS-related genes.

To investigate the functions of BcMsn2 in *B. cinerea*, RNA-seq analysis was performed using RNA samples isolated from the vegetative hyphae of the WT B05.10 and ∆*bcmsn2* from 3-day PDA cultures. Compared with the WT, 2847 differentially expressed genes (1,346 upregulated and 1,501 downregulated) were detected in the ∆*bcmsn2* mutant (Figure 6A). The upregulated genes in ∆*bcmsn2* were enriched in the cellular nitrogen compound metabolic processes, while the downregulated genes were enriched in methionine biosynthetic/metabolic processes (Figure 6B). To validate the expression pattern of ROS-related genes, eight ROS-related genes were selected, and their expression patterns were confirmed using qRT-PCR. The expression of ROS-related genes was consistent with the expected results according the RNA-Seq analysis (Figure 6C). These results indicate that BcMsn2 is involved in a number of cellular amine metabolic processes, including ROS regulation.

## 4. Discussion

The imbalance between ROS production and clearance can lead to oxidative stress, which is pervasive in the lifestyle of organisms and their interactions with their host (Saxena et al., 2016). Consequently, many organisms have developed oxidative stress response (OSR) mechanisms to scavenge elevated intracellular ROS levels (Yaakoub et al., 2022). In the yeast *S. cerevisiae*, the transcription factors Msn2 and Msn4 regulate 200 genes in response to various stresses, including heat shock, osmotic shock, low pH, sorbic acid, glucose starvation, oxidative stress, and high ethanol concentrations (Martínez-Pastor et al., 1996; Schmitt and McEntee, 1996; Gasch et al., 2000; Causton et al., 2001). Msn2 and Msn4 of *S. cerevisiae* are two C_2_H_2_ transcription factors involved in the Ras-cAMP pathway that is regulated by Ras-cAMP protein kinase A (Ras-cAMP-PKA), which activates genes containing the stress response element (STRE: CCCCT) to protect against oxidative stress and to maintain a reduced cellular redox balance (Martínez-Pastor et al., 1996; Schmitt and McEntee, 1996; Beck and Hall, 1999). Overexpression of *Msn2* increases oxidation tolerance by enhancing Msn2-mediated proline binding (Mat Nanyan and Takagi, 2020). However, we only identified one homologous gene of *Msn2/4* from *S. cerevisiae* in *B. cinerea*, which exhibits 66.6% and 64.9% identity with *Msn2* and *Msn4*, respectively. This might be attributed to functional divergence, because Msn2 and Msn4 are functionally redundant in *S. cerevisiae* (Gasch et al., 2000). Different with *S. cerevisiae* that is a single celled eukaryotic organism and has no pathogenicity to hosts, *B. cinerea* are more related to filamentous pathogenic fungi. It was reported that ∆*Bbmsn2* and ∆*Mrmsn2* showed remarkable defects in conidial yield (40% decrease) and virulence (25% decrease) in two entomopathogenic fungi *Beauveria bassiana* (Bb) and *Metarhizium robertsii* (Mr) (Liu et al., 2013). Disruption of the *MoMsn2* gene of *M. oryzae* resulted in defects in aerial hyphal growth, conidial production, and infection of host plants (Zhang et al., 2014). Similar phenotypes indicated that the function of the *Msn2* gene in filamentous fungi is conserved.

Disruption of the *BcMsn2* gene led to a significant increase in intracellular ROS levels. The involvement of BcMsn2 in the regulation of *Sod*, *Cat*, and *NoxD* genes in *B. cinerea* was confirmed through RNA-seq and qRT-PCR analyses. The downregulated genes were enriched in the methionine biosynthetic/metabolic processes. Methionine sulfoxide reductases (MsrA/Mxr1 and MsrB/Mxr2) reduce free oxidized methionine by acquiring electrons from the thioredoxin-reducing or glutathione system (García-Santamarina et al., 2013). The findings indicated that BcMsn2 might participate in oxidative stress and intracellular redox homeostasis by regulating Msr activity. Although SOD and CAT exhibit functional redundancy in *B. cinerea*, the loss of *Cat3* is not compensated for by other catalases, resulting in increased development of aerial hyphae and conidia formation in *N. crassa* (Michán et al., 2003). The NADPH oxidase (NOX) complex in the plasma membrane is a primary ROS-producing protein complex (Li et al., 2019); however, NOX enzymes are not major contributors to ROS production in *B. cinerea* (Segmüller et al., 2008). Enzymatic ROS scavenging mechanisms are vital for suppressing toxic ROS levels. These results indicated that the BcMsn2 plays an important role in maintaining the balance between oxidizing and reducing equivalents, thereby regulating the intracellular redox state. Cellular ROS concentrations exhibit circadian oscillations (Yoshida et al., 2011). BcFRQ1 functions as a core negative clock element in the circadian system, regulating the conidiation and pathogenicity of *B. cinerea* (Hevia et al., 2015). It has been demonstrated that the ROS concentration regulates the transcriptional function of WCC, a transcription factor that promotes *frq* expression in *N. crassa* (Yoshida et al., 2011). The RNA-seq results identified a related gene (Gene ID: Bcin07g04440) with downregulated expression, which might be regulated by BcMsn2 and is involved in the cross-talk between the cellular redox state and the circadian system, although further investigation is needed to enhance our understanding of this process.

In terms of growth, the *BcMsn2* knockout mutant exhibited a highly significant reduction in the hyphal growth rate, earlier conidial production and germination, but a decrease in the total amount of conidia produced, and a severe delay in sclerotium formation. Excessive ROS within ∆*bcmsn2* conidia might account for the unusually active conidia but slower growth rate. Hyperoxidant states are a primary driving force for fungal differentiation (Aguirre et al., 2006; Yoshida et al., 2011). Studies have indicated that the differentiation of lateral-chained and terminal sclerotia in *S. rolfsii* and *S. sclerotiorum* is reduced by approximately 30% when the H_2_O_2_ concentration is low (1–3 mM). By contrast, higher concentrations of H_2_O_2_ (5–8 mM) promote sclerotial differentiation (Papapostolou et al., 2014). BcNOXA and BcNOXD interact with each other, and BcNOXA is essential for the formation of sclerotia (Segmüller et al., 2008; Siegmund et al., 2015). Thus, decreased expression of *BcNoxD* might be the reason for delayed sclerotial formation in the *BcMsn2* knockout mutant.

In this article, we found knockout of *BcMsn2* resulted in a significant decrease in pathogenicity of *B. cinerea* on apple pear, and tomato fruits, and bean leave. These results indicated *BcMsn2* was also identified as an important virulence determinant, similar to its reported involvement in virulence in *M. oryzae*. *MoMsn2* is essential for sporulation and conidiophore development, and the ∆*Momsn2* mutant lost its pathogenicity (Zhang et al., 2014). When *B. cinerea* infects plants, the plants undergo a so-called ‘oxidative burst’, producing ROS as a crucial component of their defense responses (Govrin and Levine, 2000). *Sod* knockout had a suppressive effect on pathogenicity (Rolke et al., 2004). The balanced redox status maintained by the thioredoxin system is essential for the development and pathogenesis of *B. cinerea* (Viefhues et al., 2014; Liang et al., 2022). However, there is a contrary view, suggesting that ROS-degrading enzymes and other central components of cellular redox status are not essential for pathogenesis (Temme and Tudzynski, 2009).

In summary, unraveling the regulation mechanisms of BcMsn2 is of significant importance in understanding its function in *B. cinerea*. This study demonstrated that *BcMsn2* is a key gene in the regulation of intracellular redox homeostasis in *B. cinerea*. ROS derived from fungi play critical roles in various development processes, including conidia and sclerotia formation, as well as pathogenesis. Many studies have reported the close association between ROS and the circadian rhythm (Yoshida et al., 2011; Ayer et al., 2014; Hevia et al., 2015); however, limited studies have been conducted in *B. cinerea.* Further investigation will enhance our understanding of how the circadian clock modulates the pathogenic potential of this pathogen at different times of the day.

## Data availability statement

The datasets presented in this study can be found in online repositories. The names of the repository/repositories and accession number(s) can be found in the article/Supplementary material.

## Author contributions

PL: formal analysis, resources, validation, visualization and writing–review and editing. KW and JW: formal analysis, resources, validation, investigation, methodology, visualization, and writing–original draft. CX: formal analysis and investigation. SY: writing–review and editing. LM: conceptualization. HS: conceptualization, funding acquisition, methodology, project administration and writing–review and editing. All authors have read and approved the final version of the manuscript.

## Footnotes

^1^ http://fungi.ensembl.org/
