# Supplementary material for: A novel zinc finger transcription factor, BcMsn2, is involved in growth, development, and virulence in Botrytis cinerea
Source: Front Microbiol. 2023 Oct 17;14:1247072. doi: 10.3389/fmicb.2023.1247072 (PMC10616473; doi:10.3389/fmicb.2023.1247072)
Supplement: Supplementary file 1 [file Data_Sheet_1.docx]

Supplementary Material

A novel zinc finger transcription factor, BcMsn2, is involved in growth, development, and virulence in *Botrytis cinerea*

**Ping Lu^1†^, Ke Wang^1†^, Jiaqi Wang, Chunbo Xia^1^, Shu Yang^1^, Liang Ma^1*^, Haojie Shi^1*^**

**^*^Correspondence:**Liang Ma
E-mail: [liangm2008@outlook.com](mailto:liangm2008@outlook.com)
Haojie Shi
E-mail: [shj@zafu.edu.cn](mailto:shj@zafu.edu.cn)

# Supplementary Figures and Tables

**
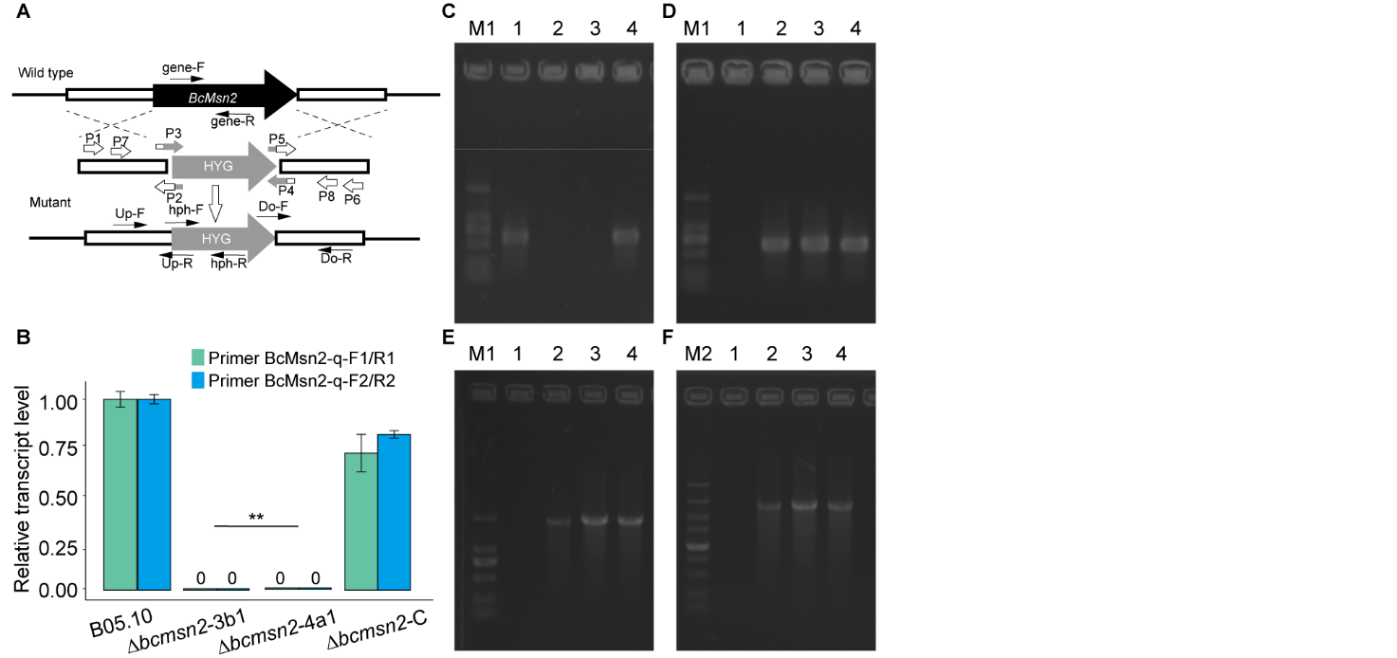
**

**Supplementary Figure 1.** Validation of gene knockouts (M1: Marker DL2000, M2: Marker DL5000, 1: B05.10, 2: *∆bcmsn2-3b1*, 3: *∆bcmsn2-4a1*, 4: *∆bcmsn2-C*). (A) Strategy for generation of *BcMsn2* gene disruption (*∆bcmsn2*) mutant strains. (B) Detection of BcMsn2 expression in the wild type (WT) (B05.10), ∆bcmsn2 and complemented (∆bcmsn2-C) strains via qRT-PCR. Error bars represent the standard deviation. (C) The gel electrophoresis of PCR validation with the primer pair check-F/check-R. (D) The gel electrophoresis of PCR validation with the primer pair hph-F/hph-R. (E) The gel electrophoresis of PCR validation with the primer pair up-F/up-R. (F) The gel electrophoresis of PCR validation with the primer pair do-F/do-R.

#
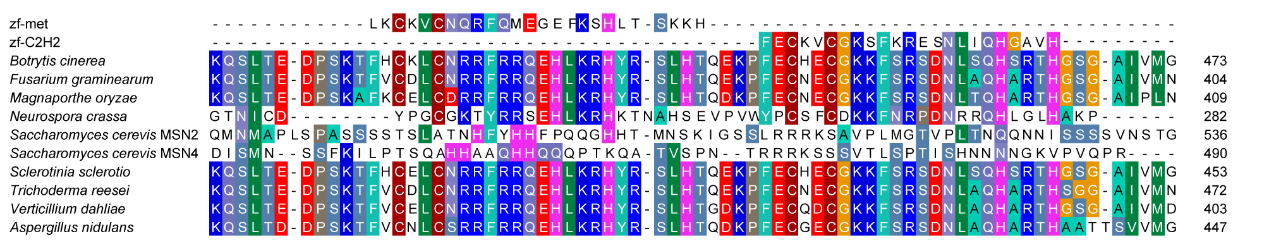


# Supplementary Figure 2. Alignment of the C_2_H_2_ zinc-finger domain and met zinc-finger domain with similar proteins from *Botrytis cinerea* (GenBank accession number XP_024546291.1), *Fusarium graminearum* (XP_011326528.1), *Magnaporthe oryzae* (XP_368743.1), *Neurospora crassa* (XP_955791.1), *Saccharomyces* *cerevisiae* (MSN2, NP 013751 and MSN4, NP 012861) *Sclerotinia sclerotiorum* (XP_001594602.1), *Trichoderma reesei* (XP_006963937.1), *Verticillium dahliae* (XP_009648969.1), *Aspergillus nidulans* (XP_659256.2).

**Supplementary Table 1.** Primers used in this study

| Primer name | Primer sequence (5'-3') | Description |
| --- | --- | --- |
| P1 | GCGAGGGAGAAAGAAAGGA | Double-joint PCR to build gene-disruption constructs. |
| P2 | AATAGGCATTGATGTGTTGACCTCCCCAAGCACAACGTACCGAT |  |
| P3 | TACGTTGTGCTTGGATACAGGAGGTCAACACATCAATGCCTATT |  |
| P4 | GTGTTCGTAAGTTTCGTAACTACTCTATTCCTTTGCCCT |  |
| P5 | AGGGCAAAGGAATAGAGTAGTTACGAAACTTACGAACAC |  |
| P6 | AGCACTGCCAACAATAGCC |  |
| P7 | AAATAGATAAAATAGATAA |  |
| P8 | AGTGAGAGGTGGATGAAGA |  |
| P9 | GAGGGAGAAAGAAAGGAAA | Construction of the complement vector p1300-BcMsn2-C |
| P10 | GAGAACAATGTGGGCAGAT |  |
| P11 | AACCATGGCCACCCTCGACGACACGGC |  |
| P12 | TAGCGGCCGCTCAGGGGCAGGGCATGCTCATG |  |
| Up-F | CAAGTTACCACGCTCCCACTCTA | Identify whether *BcMsn2* has been deleted |
| Up-R | GGGCAGTTCGGTTTCAGGCAGGT |  |
| Do-F | ATCTTAGCCAGACGAGCGGGTTC |  |
| Do-R | TTAGAGCATTTGCCTCAGGGTGC |  |
| Gene-F | GTCTTGTGAACTTCCCTTCTACCG |  |
| Gene-R | AGCCCATCATGTTGTTGCTTTCT |  |
| Hph-F | GATGTTGGCGACCTCGTATTGGG |  |
| Hph-R | TTCGACAGCGTCTCCGACCTGAT |  |
| pYES2-F | ACTATAGGGAATATTAAGCTTATGGACTCTTACGCACAAGCAA | Yeast complementation |
| pYES2-R | GCGGCCGTTACTAGTGGATCCTTACTCTGATCTCTTGCGCTTCTT |  |
| ScMsn2-F1 | CTTTGGACCTTTGGAACCATCCG |  |
| ScMsn2-R1 | CTCAAGCCTGTAGTCGCCACTTT |  |
| GAPDH-q-F | TGCCAAGAAGGTTGTTATC | Reference gene |
| GAPDH-q-R | TGTAGGTCTCGTTGTTGA |  |
| BcGOD1-q-F | GCTAGCACCGACTCTACCTT | Target gene *Bcgod1* |
| BcGOD1-q-R | CCGAATCACCTGCCTCAATG |  |
| BcSOD1-q-F | GCCCAAGGAAATGCTACTGG | Target gene *Bcsod1* |
| BcSOD1-q-R | GAGTGGACAACAACGGTACG |  |
| BcnoxD-q-F | GTGCAGTCAAGCCATTTCCA | Target gene *BcnoxD* |
| BcnoxD-q-R | AGAGCTGTCAAAGGGAGGAC |  |
| Bccgf1-q-F | AACCTCACCATACCGAAGCA | Target gene *Bccgf1* |
| Bccgf1-q-R | CGTCGCGTCCATTCTTCTTT |  |
| Bccat6-q-F | GCTGGACAGCAAGTGTTCAA | Target gene *Bccat6* |
| Bccat6-q-R | GCTGCTTGTTCGATGTCTGT |  |
| Bccat3-q-F | GAGCCACAGTACAGAAAGCG | Target gene *Bccat3* |
| Bccat3-q-R | CTCTCTCATCTCAGGCAGCA |  |
| Bcadi1-q-F | CGAGGAGAAGGTCAAGTCGT | Target gene *Bcadi1* |
| Bcadi1-q-R | ACCTAATGCGAACCCACTCA |  |
| Bc7540-q-F | GAGCTTGAAGGCGGGTATTG | Target gene *Bc7540* |
| Bc7540-q-R | GCTCTTCATTCCAGCTTCCC |  |
| BcMsn2-q-F | CCACGGAAGTGGTGCTATTG | Target gene *BcMsn2* |
| BcMsn2-q-R | CCAAGGGCTCTGATTTGCTC |  |
| BcMsn2-q-F2 | CAGCCACAAGAGACTTCTGC |  |
| BcMsn2-q-R2 | TGCAAAGCTTGCAGTGGAAT |  |
| BcMsn2-q-F3 | TCTTACGCACAAGCAATGGG |  |
| BcMsn2-q-R3 | GCTGCGAAGACGAAGAGTTT |  |

**Supplementary Table 2.** Information of Illumina RNA-Seq data used in this study

| Type of data | Sample name | Sample description | Reads length | Reads count | Mapping rates | Mapped reads | SRA Run | Reference |
| --- | --- | --- | --- | --- | --- | --- | --- | --- |
| RNA-Seq | WT-1 | wild-type mycelium culture in PDB for 3 days replate 1 | 1×75 bp | 24,134,648 | 89.49% | 21,598,096 | SRR23730095 | This study |
| RNA-Seq | WT-2 | wild-type mycelium culture in PDB for 3 days replate 2 | 1×75bp | 24,051,581 | 89.58% | 21,545,406 | SRR23730094 | This study |
| RNA-Seq | BcMsn2-1 | *BcMsn2* gene deletion mutant mycelium culture in PDB for 3 days replate 1 | 1×75 bp | 24,134,302 | 93.26% | 22,507,650 | SRR23730093 | This study |
| RNA-Seq | BcMsn2-2 | *BcMsn2* gene deletion mutant mycelium culture in PDB for 3 days replate 2 | 1×75 bp | 24,134,113 | 90.99% | 21,959,629 | SRR23730092 | This study |
